# Educational differences in self-rated physical fitness among Finns

**DOI:** 10.1186/1471-2458-13-163

**Published:** 2013-02-22

**Authors:** Kaisa R Pulkkinen, Tomi Mäkinen, Heli Valkeinen, Ritva Prättälä, Katja Borodulin

**Affiliations:** 1 Department of Chronic Disease Prevention, National Institute for Health and Welfare, PO Box 30, FIN-00271, Helsinki, Finland; 2 Department of Health, Functional Capacity and Welfare, National Institute for Health and Welfare, PO Box 30, FIN-00271, Helsinki, Finland; 3 Department of Lifestyle and Participation, National Institute for Health and Welfare, PO Box 30, FIN-00271, Helsinki, Finland

**Keywords:** Physical fitness, Physical activity, Socioeconomic position, Public health, Health

## Abstract

**Background:**

The high educated live longer and healthier lives when compared to the low educated. Physical fitness as a health indicator reflects the level of physical activity along with other health-influencing factors such as obesity, smoking, chronic diseases and individual training effects. Studies support that self-rated physical fitness correlates with objectively measured physical fitness well. However, the educational differences in self-rated physical fitness are not known.

**Methods:**

Our aim was to study educational differences in self-rated physical fitness in Finnish population. The data were collected in 2007 for a cross-sectional population based National FINRISK Study. The analyzed data included 2722 men and 3108 women aged 25 to 74 years. Statistical method was ordinal logistic regression.

**Results:**

Longer educational career was associated with better self-rated physical fitness. The educational differences in self-rated physical fitness were largely explained by health behavior. Leisure-time physical activity explained fully and body mass index partly the educational differences in self-rated physical fitness among men. The combination of body mass index, history of chronic diseases and smoking explained the differences fully among men and partly among women. Leisure-time, occupational and commuting physical activities, body mass index, history of chronic diseases and smoking together explained all educational differences in self-rated physical fitness among both genders.

**Conclusions:**

Although educational differences in self-rated physical fitness were found, they were explained by health behavior related factors. Leisure-time physical activity offered the strongest single explanation for the educational differences in self-rated physical fitness. Thus, possibilities for leisure-time physical activity should be increased especially among the low educated.

## Background

Socioeconomic position (SEP) reflects individual’s social and economic location in the structures of society. Karl Marx and Max Weber created the basics for the understanding of socioeconomic differences [1] and both traditions contributed to the modern research on health and socioeconomic circumstances. The aspects of wealth and working conditions can be seen largely of Marxian and the aspects of non-material resources and inequalities more of Weberian heritage. In this article the social stratification is described with SEP, since it reflects both Marxian and Weberian traditions [2].

Physical fitness (PF) is “a set of health or skill related attributes that people have or can achieve” [3]. A person with good PF integrates the use of the attributes effectively to reach an optimal performance. As a result PF enables “to carry out daily tasks with vigour and alertness without undue fatigue and ample energy to enjoy leisure-time pursuits and meet unforeseen emergencies” and “to achieve the optimal quality of life” [4] Physical activity (PA) is defined as “any bodily movement produced by skeletal muscles, that results in energy expenditure” [3].

Those in higher SEP have longer and healthier lives compared to those in lower SEP [1,5,6]. PA is a well-known contributor to good health [7-9] and the main way to improve PF [3,4,8,10], when performed regularly in adequate amounts and proper intensities [8,11]. In addition to PA, PF is also influenced by energy balance, smoking, chronic diseases [10] as well as individual training effects and characteristics that includes genetic variability [12-15]. PF enhancing health behavior is more common in higher than lower socioeconomic groups. For example, those in higher SEP have higher PA levels [16-22], less obesity [23-25], smoke less [24,26,27] and have less chronic diseases [28,29] than those in lower SEP.

Health-related PF is a combination of cardiovascular endurance, body composition, muscular strength, muscular endurance and flexibility [3]. Good PF is inversely associated with mortality and chronic morbidity [10,12]. It has associations also with better lipid and lipoprotein profile, lower blood pressure, better body composition, lower inflammation levels, improved autonomic nervous system [10] and improved insulin sensivity [10,30]. To have an overall picture of health-related PF, all components should be measured [4]. The use of self-rated PF (SRPF) might be a useful option in population based studies, since measuring objectively all PF dimensions is time consuming and expensive. Studies support that SRPF correlates with objectively measured PF rather well [31-33].

In this study, we used birth cohort adjusted years of education to indicate SEP. Education is widely used in health-related research because it reflects both material and immaterial resources such as skills, knowledge, attitudes and values [1,2]. Education is also an important indicator of life course influences as childhood circumstances often influence adult education levels [2]. Furthermore, education is a strong determinant of occupational status, working conditions and income [1,2,34]. It is usually also more reliably reported than e.g. income levels [2].

While the educational differences in PA have been reported in detail [16,20,21], associations of education and PF have remained largely unexplored. Our purpose was to explore whether SRPF is associated with age and education and to examine whether PA, body mass index (BMI), history of chronic diseases and smoking contribute to the possible association of education and SRPF. We assumed that the young report more good PF levels than the elderly and that those with less education report more poor PF levels than those with longer educational career [35]. We assumed also that leisure-time PA (LTPA) and BMI would contribute to the educational differences in SRPF, because low LTPA levels [17-19] as well high BMI [23] are more common in lower than higher educational groups, and both of them are known to correlate with SRPF [36].

## Methods

### Design and data collection

The data were collected in spring 2007 for a cross-sectional population based National FINRISK Study [37] that was conducted by the former National Public Health Institute, currently The National Institute for Health and Welfare. A stratified random sample was drawn from the population register with stratifications of sex, 10-year age groups and five geographical areas. Data were collected via self-administered questionnaires and health examinations carried out by trained nurses. The total sample size was 10000 of which 6258 (62.6%) persons filled out the questionnaire and participated in the health examination. Participants were excluded, if they had missing data. The final data included 2722 men and 3108 women (total of 5830) aged 25 to 74 years. The entire study protocol followed the WHO MONICA protocol [38] and later the recommendations of the European Health Risk Monitoring Project [39]. The Ethics Committee of the Hospital District of Helsinki and Uusimaa approved the study protocol and the participants provided a written consent.

### Measurements

#### Self-rated physical fitness and education

The dependent variable was SRPF. PF was measured with a question of “How do you consider your current physical fitness?” The answer categories were very good, quite good, fair, quite bad and very bad. The two last categories were combined for the analyses because of the small sample size in the last category. The education level was assessed by a question of “How many years have you attended school and studied full-time (basic levels included)?” The birth cohort adjusted education thirds were labeled low, middle and high.

#### Physical activity

Three modes of PA were inquired via questionnaire. Commuting PA (CPA) was measured with a question of “How many minutes do you walk, ride on bicycle or otherwise exercise to get to work?” Instructions guided to take into account both travelling to and from work. The answer categories were combined for the analyses into three groups: 30 minutes or more in a day, less than 30 minutes a day or no CPA at all. The question of LTPA combined the type, intensity and amount of LTPA. The question was “How much do you exercise and stress yourself physically in your leisure time?” The additional instructions guided to choose the average LTPA level, if the activity varies much according to different seasons. The answers were categorized in to three categories of high, medium and low activity levels. Occupational PA (OPA) was measured with a question: “How demanding is your work physically?” Instructions guided to choose sedentary work, if the person is not working at all. The answers were categorized into three groups: heavy, moderate and light work.

#### Confounding factors

The information about age and gender was received from the population register. Age was used in the analyses as a continuous variable. The height and weight were measured in the health examination. For BMI the weight in kilograms was divided by squared height in meters (kg/m^2^). The questionnaire included questions about chronic diseases that had been diagnosed by a physician. Chronic diseases comprised asthma, cancer, cardiovascular diseases (myocardial infarction, stroke, cerebral hemorrhage, obstruction of a cerebral vessel, coronary bypass surgery, coronary angioplasty, hypertension, cardiac insufficiency, angina pectoris), diabetes, chronic obstructive pulmonary disease, rheumatoid arthritis, degenerative arthritis of the back or other joint or back related chronic diseases. The participants were divided into two categories: those who reported at least one chronic disease and others. Smoking was assessed in the questionnaire by questions of how often and how much the participant smoked and when was the last time he or she had smoked. The answers were categorized into three groups: non-smokers, former smokers and daily smokers. If participants reported occasional smoking that had never been regular, they were categorized into non-smokers.

#### Statistical analyses

The analyses were performed by ordinal logistic regression with logit -link function. The statistical software was PASW/SPSS Statistics 18 for Windows (Armonk, NY, USA). Since PASW/SPSS does not print odds ratios (OR) they were calculated in Microsoft Word Excel 2003 for Windows (Redmond WA, USA). The assumptions for the analysis were tested and fulfilled. The parallel lines assumption was tested by a random sample of 3% of the data [40]. The results are reported in ORs separately for women and men.

## Results

### Age and education associations with self-rated physical fitness

Mean age was 49.7 years (Standard deviation (SD) 13.9) among women and 51.2 years (SD 13.8) among men (Table 1). Age had an inverse association with SRPF in the unadjusted analyses. The elderly were more likely to report poor PF when compared to younger adults (Table 2). Education was directly associated with SRPF in the unadjusted and age adjusted analyses (Tables 2 and 3). Those in the high education third reported better PF than those the in low education third (Table 3).

**Table 1:** Descriptive characteristics of the participants (n = 5830)

		**Women (n = 3108)**		**Men (n = 2722)**	
		**% or mean**	**(n or SD)**	**% or mean**	**(n or SD)**
**Age group**	25–34	18.1	(412)	15.1	(562)
%	35–44	19.9	(509)	18.7	(619)
	45–54	21.5	(564)	20.7	(668)
	55–64	20.9	(624)	22.9	(649)
	65–74	19.6	(613)	22.5	(610)
**Self-rated physical fitness**	Poor	11.9	(370)	12.2	(333)
%	Fair	40.1	(1245)	40.2	(1095)
	Good	42.5	(1320)	39.6	(1079)
	Very good	5.6	(173)	7.9	(215)
**Education thirds**^**1**^	High	16.88	(3.08)	16.29	(2.79)
mean years (SD)	Middle	12.68	(2.71)	11.40	(2.42)
	Low	9.97	(2.50)	9.01	(2.12)
**Commuting PA**	≥ 30 min	16.7	(520)	10.5	(285)
%	< 30 min	27.1	(841)	25.3	(690)
	Inactive	56.2	(1747)	64.2	(1747)
**Leisure time PA**	High	25.5	(792)	28.3	(770)
%	Medium	54.9	(1706)	51.1	(1391)
	Low	19.6	(610)	20.6	(561)
**Occupational PA**	Heavy	16.6	(516)	28.6	(778)
%	Moderate	27.3	(850)	20.3	(553)
	Light	56.0	(1742)	51.1	(1391)
**BMI**	< 25	44.4	(1381)	29.6	(806)
%	25–29.9	33.0	(1026)	48.7	(1326)
	≥ 30	22.6	(701)	21.7	(590)
**Chronic Diseases**^**2**^	No	58.1	(1806)	53.8	(1464)
%	Yes	41.9	(1302)	46.2	(1258)
**Smoking**	Never	63.5	(1973)	43.6	(1188)
%	Former	19.6	(609)	32.2	(876)
	Daily	16.9	(526)	24.2	(658)

^1)^ education represented in mean years (standard deviation in parentheses).

^2)^ asthma, cancer, cardiovascular diseases, diabetes, chronic obstructive pulmonary disease, rheumatoid arthritis, degenerative arthritis of the back or other joint or back related chronic diseases.

**Table 2:** Crude odds ratios (OR) for poor self-rated physical fitness

**Variables**		**Women (n = 3108)**				**Men (n = 2722)**			
		**n**	**Poor fitness n (%)**	**OR**	**95% CI**	**n**	**Poor fitness n (%)**	**OR**	**95% CI**
**Age group** (years)	25–34	562	49 (8.7)	1.00	(ref)	412	25 (6.1)	1.00	(ref)
	35–44	619	74 (12.0)	1.29	1.04–1.59	509	56 (11.0)	1.28	1.01–1.64
	45–54	668	94 (14.1)	1.54	1.24–1.90	564	76 (13.5)	1.59	1.25–2.02
	55–64	649	80 (12.3)	1.73	1.39–2.13	624	99 (15.9)	1.97	1.56–2.49
	65–74	610	73 (12.0)	1.94	1.57–2.42	613	77 (12.6)	1.96	1.55–2.48
**Education thirds**	High	1079	109 (10.1)	1.00	(ref)	973	108 (11.1)	1.00	(ref)
	Middle	1036	117 (11.3)	1.23	1.04–1.44	963	126 (13.1)	1.26	1.06–1.49
	Low	993	144 (14.5)	1.54	1.31–1.81	786	99 (12.6)	1.39	1.17–1.65
**Commuting PA**	≥ 30 min	520	30 (5.8)	1.00	(ref)	285	22 (7.7)	1.00	(ref)
	< 30 min	841	99 (11.8)	1.50	1.22–1.84	690	69 (10.0)	1.07	0.82–1.38
	Inactive	1747	241 (13.8)	1.97	1.63–2.37	1747	242 (13.9)	1.48	1.18–1.88
**Leisure time PA**	High	792	9 (1.1)	1.00	(ref)	770	14 (1.8)	1.00	(ref)
	Medium	1706	158 (9.3)	4.18	3.50–4.99	1391	139 (10.0)	5.02	4.18–6.03
	Low	610	203 (33.3)	17.82	14.17–22.44	561	180 (32.1)	14.72	11.67–18.58
**Occupational PA**	Heavy	516	52 (10.1)	1.00	(ref)	778	65 (8.4)	1.00	(ref)
	Moderate	850	78 (9.2)	0.91	0.74–1.12	553	47 (8.5)	0.99	0.80–1.20
	Light	1742	240 (13.8)	1.37	1.14–1.65	1391	221 (15.9)	1.53	1.29–1.80
**Body mass index** (kg/m^2^)	< 25	1381	77 (5.6)	1.00	(ref)	806	51 (6.3)	1.00	(ref)
	25–29.9	1026	110 (10.7)	2.28	1.95–2.66	1326	136 (10.3)	1.79	1.52–2.12
	≥ 30	701	183 (26.1)	6.00	4.99–7.20	590	146 (24.7)	4.31	3.51–5.30
**Chronic diseases**^**1)**^	No	1806	138 (7.6)	1.00	(ref)	1464	97 (6.6)	1.00	(ref)
	Yes	1302	232 (17.8)	2.60	2.27–2.99	1258	236 (18.8)	2.61	2.26–3.01
**Smoking**	Never	1973	213 (10.8)	1.00	(ref)	1188	101 (8.5)	1.00	(ref)
	Former	609	77 (12.6)	1.04	0.88–1.24	876	128 (14.6)	1.61	1.37–1.90
	Daily	526	80 (15.2)	1.32	1.10–1.58	658	104 (15.8)	1.87	1.56–2.23

Self-rated physical fitness categories are poor, fair, good and very good.

*PA* = physical activity, *CI* = confidence interval.

^**1)**^ asthma, cancer, cardiovascular diseases, diabetes, chronic obstructive pulmonary disease, rheumatoid arthritis, degenerative arthritis of the back or other joint or back related chronic diseases.

**Table 3:** Age adjusted associations of education and poor self-rated physical fitness among women and men

	**Education thirds**									
	**Women**					**Men**				
	**High**	**Middle**		**Low**		**High**	**Middle**		**Low**	
**Models and adjustments**	**OR**	**OR**	**(95% CI)**	**OR**	**(95% CI)**	**OR**	**OR**	**(95% CI)**	**OR**	**(95%CI)**
**Model 1: Age**	1.00	1.23	(1.05–1.45)	1.57	(1.33–1.84)	1.00	1.26	(1.07–1.48)	1.42	(1.19–1.69)
**Model 2: Physical activity (PA)**										
**2a)** Commuting PA (CPA)	1.00	1.21	(1.03–1.43)	1.56	(1.32–1.83)	1.00	1.23	(1.04–1.46)	1.38	(1.16–1.65)
**2b)** Leisure-time PA (LTPA)	1.00	1.21	(1.02–1.43)	1.37	(1.16–1.62)	1.00	1.01	(0.85–1.19)	0.99	(0.83–1.19)
**2c)** Occupational PA (OPA)	1.00	1.25	(1.07–1.47)	1.62	(1.37–1.91)	1.00	1.36	(1.15–1.61)	1.57	(1.31–1.89)
**2d)** CPA + LTPA + OPA	1.00	1.21	(1.02–1.43)	1.39	(1.17–1.65)	1.00	1.08	(0.90–1.28)	1.10	(0.90–1.33)
**Model 3: Health & lifestyle**										
**3a)** Body Mass Index (BMI)	1.00	1.18	(1.00–1.39)	1.36	(1.15–1.60)	1.00	1.13	(0.95–1.34)	1.29	(1.08–1.54)
**3b)** Chronic diseases ^1^	1.00	1.18	(1.01–1.39)	1.48	(1.25–1.74)	1.00	1.21	(1.30–1.43)	1.34	(1.12–1.60)
**3c)** Smoking	1.00	1.22	(1.04–1.43)	1.50	(1.27–1.77)	1.00	1.20	(1.01–1.42)	1.28	(1.08–1.53)
**3d)** BMI + Chronic diseases + Smoking	1.00	1.13	(0.96–1.33)	1.25	(1.05–1.48)	1.00	1.06	(0.90–1.26)	1.14	(0.95–1.36)
**Model 4: Physical activity + Health & Lifestyle **(CPA + LTPA + OPA + BMI + Chronic diseases + Smoking)	1.00	1.13	(0.95–1.34)	1.17	(1.00–1.42)	1.00	0.97	(0.81–1.16)	0.97	(0.78–1.18)

*OR* = odds ratio, *CI* = confidence interval.

^1)^ asthma, cancer, cardiovascular diseases, diabetes, emphysema, rheumatoid arthritis or other joint or back related chronic diseases.

### The mediating effect of physical activity

LTPA had a strong gradient association with SRPF in the unadjusted analyses (Table 2). The odds of reporting poor PF was more than 17 times (OR 17.82, 95% Confidence Interval (CI) 14.17–22.44) more likely for women with low LTPA than those with high LTPA levels. The strong association was also seen among men (OR 14.72, 95%CI 11.67–18.58). Even with medium LTPA levels the odds of reporting poor SRPF was higher among women (OR 4.18 95%CI 3.50–4.99) and men (OR 5.02 95%CI 4.18–6.03), when compared to those with high LTPA levels. Also those with low level of OPA and CPA reported poorer PF levels than their physically most active counterparts.

In the adjusted analyses LTPA explained the educational differences in SRPF among men. However, among women the educational differences remained statistically significant (Table 3). OPA increased somewhat the educational differences in SRPF between the low and high education thirds. The combined adjustments for all modes of PA did not offer any additional information for the only LTPA adjusted model.

### The mediating effects of body mass index, history of chronic diseases and smoking

Average BMI was 26.7 kg/m^2^ (SD 5.4, range 16.4–53.1 kg/m^2^) among women and 27.4 kg/m^2^ (SD 4.2 range 16.0–63.3 kg/m^2^) among men (Table 1). High BMI increased the odds for poor SRPF (Table 2). The likelihood to report poor SRPF was higher among overweight men (OR 1.79, 95%CI 1.52–2.12) and women (OR 2.28, 95%CI 1.95–2.66) and among obese men (OR 4.31, 95%CI 3.51–5.30) and women (OR 6.0, 95%CI, 4.99–7.20). Those with chronic diseases were more likely to report poor PF than the healthy. Regular daily smoking was associated more often with poor SRPF when compared to non-smokers. Among men also former regular smoking increased the odds for poor SRPF, but among women the association was statistically non-significant.

BMI explained the educational differences in SRPF between the middle and high educational thirds among men (Table 3). However, the educational difference remained statistically significant between the low and high educational thirds among men and between all educational thirds among women. Chronic diseases or regular smoking did not contribute statistically significantly to the age adjusted educational differences in SRPF although minor decreases were seen especially when adjusted for chronic diseases. The combination of BMI, chronic diseases and smoking explained all educational differences among men and the educational difference of the high and middle educational thirds among women.

### The final model

The combination of age, three types of PA, BMI, smoking status and history of chronic diseases status explained all educational differences in SRPF among both genders (Table 3). Compared to the high educated, the odd ratios for the middle and low educated women were 1.13 (95% CI 0.95–1.34), 1.17 (95% CI 1.00–1.42, p = 0.057) and for middle and low educated men 0.97 (95% CI 0.81–1.16), 0.97 (95% CI 0.78–1.18), respectively.

### Interactions

The variables LTPA, CPA, BMI and education had statistically significant interactions with each other. The directions of the associations were same as in main effects: less PA, higher BMI and lower education were associated with poorer SRPF.

## Discussion

Our purpose was to explore whether SRPF is associated with age and education and to examine whether PA, BMI, history of chronic diseases and smoking contribute to the possible association of education and SRPF. Our large population based data supported our hypotheses and suggested that age was inversely associated with SRPF. Education was directly associated with SRPF, and these educational differences were explained by health behavior related factors. LTPA, the strongest single contributor, explained fully and BMI partly the educational differences in SRPF among men. The combination of BMI, history of chronic diseases and smoking explained all educational differences in SRPF, except for the low educated women. The combination of age, three modes of PA, BMI, smoking status and history of chronic diseases explained all educational differences in SRPF among both genders. The strengths of this study are a representative population sample and education as the main indicator of SEP. The response rate of FINRISK 2007 study was 62.6%. The participation rate was acceptable, but those who do not participate are known to be more often less educated [41,42]. If the participation rate would have been higher and thus the proportion of the less educated would have been higher, it is possible that the educational differences in SRPF would have been even more pronounced than reported.

According to our study, the strongest single contributor for the educational differences in SRPF was LTPA. Also previous studies support that LTPA levels are higher among the high educated when compared to the low educated [16,18,21,24]. Good PF is often due to high level of PA [3,4,10]. In addition to PA, material welfare and social influences may explain the educational differences in SRPF. Better material circumstances increase the possibility and variety to participate in LTPA among the high educated when compared to the low educated [1,2,34,43]. Moreover, social appreciation and support possibly gained through high education is likely to increase the participation in LTPA [44,45]. The low educated may also live in more deprived neighborhoods, where positive LTPA may be restricted by inadequate sports facilities, feelings of unsafe [43] or social control and norms [44].

Apart from LTPA, we found that other modes of PA could not explain the educational differences in SRPF. However, the questionnaire measured only the amount but not the intensity of CPA. It is therefore possible that all CPA reported was not contributing to PF. For example, CPA may be performed out of necessity instead of own motivation if other types of commuting are not possible. This is likely at least, if the household cannot afford to buy and maintain a car, and the public transportation services are inadequate in the neighborhood. If CPA is performed out of necessity, the fitness promoting intensities may even be avoided especially, if the working place lacks proper changing rooms and showering facilities. In this assumption the socioeconomic differences could exist if not in the amount but in the intensity of CPA.

In our study, BMI explained educational differences in SRPF only partly and only among men. It has been studied that BMI correlates with SRPF, but when adjusted with LTPA, the association between BMI and SRPF disappears [36]. This could indicate that LTPA would be a mediating factor between BMI and SRPF at least for middle-aged male employees. It is also established that LTPA would mediate the association of BMI and SEP for women, but not for men [46]. Thus it seems that LTPA mediates the health behavior related factors which BMI influence, but mediating pathways might be different among men and women. This difference may offer some explanations for the fact that we found partial mediating effects with BMI among men but not among women, and that LTPA explained educational differences in SRPF among men but not among women.

We did not expect that the explaining factors would differ between women and men. The gender difference in the explaining factors may arise either from the different views of SRPF among women and men or from the gender difference within the actual health behavior. To our knowledge, it is not known how individuals estimate their PF. However, it is likely that they compare their abilities with a reference group, which may consist of co-workers, friends, neighborhood inhabitants, own previous PF or others of the same gender. In this respect, individual SRPF may vary a great deal according to the reference group that has been chosen. For example, comparing PF with co-workers in physically straining work is likely to produce different estimations than if the reference groups would consist mainly of those in light work. Also previous severe illnesses of very high sport participation in past may affect individual estimates of individual PF.

We assume that conceptual differences may also contribute to PF estimations. For example, men may consider PF more as physical performance and women more as general well-being. If it is assumed that men consider PF more as physical performance and women as general well-being, it is also likely that men perform LTPA more often PF oriented and women just for having fun and enjoyment. Interestingly, according to our results, (slightly) a higher proportion of men than women considered their fitness level to be poor even when they reported high or medium LTPA levels (Table 2). Thus it is likely that some of the gender differences in our findings can be explained by conceptual differences in SRPF. It is most likely that the concept of SRPF vary also according to other attributes than gender, for example according to age or educational level.

For the future studies, more specific validation is needed to clarify the correspondence of self-rated and objectively measured PF. Also the associations of SRPF and other health measures such as self-rated health and functional ability need to be explored as well as the association of SRPF and other SEP indicators, such as income and occupation.

## Conclusion

Educational differences in SRPF were found, but they could be explained by health behavior related factors. LTPA offered the strongest single explanation for the educational differences in SRPF. Enhancing LTPA among the low educated could improve public health and decrease the health inequalities between the educational groups.

## Abbreviations

SEP: Socioeconomic position; PA: Physical activity; PF: Physical fitness; SRPF: Self-rated physical fitness; BMI: Body mass index; LTPA: Leisure-time physical activity; CPA: Commuting physical activity; OPA: Occupational physical activity; SD: Standard deviation; OR: Odds ratio; CI: Confidence interval

## Competing interests

The authors declare no competing interests.

## Authors’ contributions

KP carried out the analyses and drafted the manuscript with the support of KB. All authors participated in planning of the manuscript and commented the different manuscript versions of the article. In addition, TM provided his expertise in statistical methods, KB and HV their expertise in physical fitness and RP her expertise in health behavior in general and among subgroups. All of the authors have approved the final version of the manuscript.

## Pre-publication history

The pre-publication history for this paper can be accessed here:

http://www.biomedcentral.com/1471-2458/13/163/prepub
